# Hearing aids: indications, technology, adaptation, and quality control

**DOI:** 10.3205/cto000147

**Published:** 2017-12-18

**Authors:** Ulrich Hoppe, Gerhard Hesse

**Affiliations:** 1 Section of Audiology, Department of Otolaryngology, Head and Neck Surgery, University of Erlangen, Germany; 2 Tinnitus Department, Hospital of Bad Arolsen, University of Witten-Herdecke, Germany

**Keywords:** hearing loss, hearing aid, rehabilitation, audio therapy, tinnitus

## Abstract

Hearing loss can be caused by a number of different pathological conditions. Some of them can be successfully treated, mainly by surgery, depending on the individual’s disease process. However, the treatment of chronic sensorineural hearing loss with damaged cochlear structures usually needs hearing rehabilitation by means of technical amplification. During the last two decades tremendous improvements in hearing aid technology led to a higher quality of the hearing rehabilitation process. For example, due to sophisticated signal processing acoustic feedback could be reduced and hence open fitting options are available even for more subjects with higher degrees of hearing loss. In particular for high-frequency hearing loss, the use of open fitting is an option. Both the users’ acceptance and the perceived sound quality were significantly increased by open fittings.

However, we are still faced with a low level of readiness in many hearing impaired subjects to accept acoustic amplification. Since ENT specialists play a key-role in hearing aid provision, they should promote early hearing aid rehabilitation and include this in the counselling even in subjects with mild and moderate hearing loss. Recent investigations demonstrated the benefit of early hearing aid use in this group of patients since this may help to reduce subsequent damages as auditory deprivation, social isolation, development of dementia, and cognitive decline. For subjects with tinnitus, hearing aids may also support masking by environmental sounds and enhance cortical inhibition.

The present paper describes the latest developments of hearing aid technology and the current state of the art for amplification modalities. Implications for both hearing aid indication and provision are discussed.

## 1 Introduction to device-related hearing rehabilitation – lack of causal treatment modalities of inner ear hearing loss

Up to now it is not possible to curatively treat the causes of (chronic) inner hear hearing loss and the associated impairment regarding communication and thus also reduced quality of life and participation in social life. So rather rehabilitative approaches are available for therapy.

Hearing loss can be compensated, even corrected, but only rarely hearing can be completely restored. At best, recovery – often also complete – is possible after an acute stage of sudden hearing loss or after noise-induced trauma. However, it can only be expected if therapeutic steps are undertaken to enhance this recovery; the current data situation does not confirm this assumption [1]. So the decision to start drug therapy is only suitable in the acute stage; there are no possibilities to causally treat chronic inner ear disease with pharmaceutics. Furthermore, the question of how long such an “acute phase” would last, cannot be answered with sufficient evidence; in the first 3 months, however, improvements are possible und thus justify therapeutic attempts. Afterwards, they are useless with regard to regeneration or recovery of existing hair cell damage.

According to a current review article from Singapore [2], causal treatment of hearing loss would only be possible by gene therapy, either by direct administration of genes for regeneration of the sensory epithelium, by application of stem cells, or by drugs that induce the growth of hair cells. Hereby, gene therapy would have to be individual, in particular, target cells would have to be determined, i.e. those cells that are necessary for control and regulation of the regeneration of the inner ear. Possibly, gene therapy may also be combined with cochlear implantation, e.g. for targeted stimulation of the spiral ganglia cells. It is important to achieve a more exact understanding of the molecular correlations that cause cellular trauma or death [3]. Currently, the application of neurotrophins is investigated in animal experiments, again to support CI interventions and promotion of the neurite growth [4]. Those therapeutic approaches, however, may not yet be applied in humans.

A conceivable option would also be a therapy of inner ear hearing loss with stem cells in order to increase the population of functional neurons that are often reduced in cases of longer-lasting hearing loss and only allow moderately successful cochlear implantation [5].

Only for the application of stem cells in the treatment of hearing loss, a review article [6] found 455 single article, 48 of which could be evaluated. However, therapeutic options currently do not result from those publications. 

In their current review of 2014, Géléoc and Holt [7] try a very optimistic prognosis and state that the possibilities of genetic restoration and regeneration of the inner ear have progressed enormously. According to their point of view, it is known especially from cochlear implantation that many deficits can be compensated by cortical plasticity and learning effects. However, in the outlook of their publication, they mention that the newly created hair cells still would have to take over the functions at the defined location of the basal membrane and that this is a great challenge.

### Conclusion

Soberly speaking, most of the articles on gene therapy only describe possibilities and discuss research projects. Actually, the development of therapeutic approaches that might be applied protectively for noise exposition or support cochlear implantation, seems to be more probable than the possibility to completely replace the damaged structures of the inner ear.

### 1.1 Rehabilitative measures

Even if causal therapy of inner ear hearing loss cannot be expected for the near future, there are several possibilities have been developed to compensate hearing loss and they have been definitely improved due to new technologies. Furthermore, there are extensive publications on hearing aids (reviews in [8], [9]) and numerous articles on cochlear implant.

Still 15 or 20 years ago, most of the adapted (better) hearing aids were products to be used in the ear with the claim to be mostly invisible and to thus avoid stigmatization. Nowadays, modern hearing aids are openly adapted behind-the-ear versions with a visible device and an integrated microphone. This makes wearing hearing aids more comfortable because the “occlusion effect” caused by the closure of the auditory canal, is avoided and especially the high frequencies are effectively amplified. 

Since most of the inner ear hearing impairments, especially in older patients, start in the high-frequency ranges, an effective high-quality regeneration is possible. Only in cases of more severe hearing loss or hearing impairment in the deep frequencies, individually adapted otoplastics are required to achieve sufficient amplification. However, even for those devices the wearing comfort can be significantly increased via vents due to available modern noise suppression.

**Figure 1:**
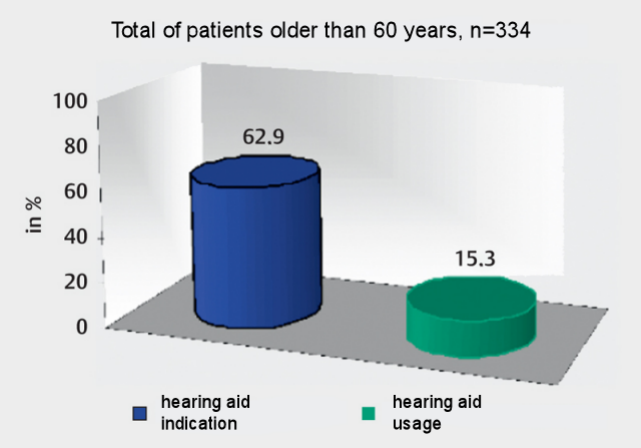
Relationship between hearing aid indication and hearing aid usage [42]

Nonetheless, the care for hearing impaired people is rather poor – and not only in Germany (Figure 1 (Fig. 1)). Often, really high-quality devices can only be obtained by paying additional sums although the minimum contributions and minimum requirements to hearing aids have been increased and improved by the statutory health insurances in 2014. But it is still in particular the stigmatization and the poor reputation of hearing aids that keeps hearing impaired people from accepting an adequate hearing systems. According to current percentages, only 15% of actually hearing impaired people (only in Germany those are 15 million candidates!) wear hearing aids.

Nevertheless, hearing aids should be recommended and prescribed as early as possible in order to prevent deprivation of the hearing pathway and especially of the auditory cortex (for further information see chapter 12. Excursion: Hearing loss and dementia).

In the context of a large epidemiologic study in the USA (Beaver Dam Offspring Study) performed between 2005 and 2008, 3285 patients with an average age of 49 years were questioned about hearing aids and their usage [10]: 34% had a hearing problem and had undergone hearing test. Only 22.5% of the people with moderate to severe hearing loss wore hearing aids.

A very interesting study from Giessen [9] evaluated 664 questionnaires. The study was performed as a multicenter project in cooperation with 79 hearing care professionals. The questionnaires contained 20 items on the consumer benefit of modern hearing aids; they were answered by 421 first users and 243 experienced hearing aid wearers. The benefit was significantly higher for hearing impaired people with modern hearing devices compared to older hearing aids from the past. Most significant was the gain with regard to hearing of natural sounds, poorest with regard to the capacity to ignore background noise.

### 1.2 Binaural provision of hearing aids is the standard treatment

In a study from Florida, a new test battery could again demonstrate the superiority of binaural provision of hearing aids that – according to the applicable regulation from 2012 – is the general rule in Germany [11]. Signals and background noises were provided in several trial arrangements and evaluated with altering laterality and with different hearing aid options (left, right, binaural). In 80% of those 20 hearing impaired test persons, binaural care was superior, just some older patients preferred unilateral hearing aids [12].

In an evaluation study from Israel on the use of hearing aids, the superiority of modern digital hearing devices was described. 177 hearing impaired adults were provided with such a hearing system and questioned about their satisfaction. 74% participated in the interviews, 83% of them used their hearing aids regularly, 17% did not. Among the users, 92% were satisfied. The patients who did not wear their hearing aids, complained about too high amplification of background noises and only poor hearing gain. The authors require better information about a usage of the hearing aids as regular as possible and training of special hearing situations [13]. 

Furthermore, good information improves the acceptance of hearing aids, as confirmed by an investigation from Portland, Oregon. For this study, 60 first users of hearing aids were randomly assigned to 3 groups. They received information and consultation of different intensity and elaboration. The more intensive the consultation was with regard to specific hearing situations, the better was the acceptance of the hearing device. It was also important to discuss unrealistic expectations to hearing aids and to emphasize positive aspects [14].

### Conclusion

Patients should be informed very realistically with regard to what can be achieved with hearing aids and where the limits are. Hearing and audio-therapy should ideally accompany hearing aid provision and train specific hearing situations with the hearing device [15].

## 2 Indications based on current guidelines of hearing aid prescription

For the statutory health insurances, the indications of hearing aids is based on the respective paragraph dated April 1, 2012, of the Federal Ministry of Health, that was lastly revised on December 17, 2015. According to those regulations

- surgical hearing improvement should not be possible,
- the tone audiometric hearing loss in the better hearing ear should be at least 30 dB in at least one of the test frequencies between 500 and 4000 Hz, and
- speech audiometry should reveal an understanding in the better hearing ear with headphones in the Freiburg monosyllabic test of not more than 80% at 65 dB,
- the patient should be sufficiently cooperative.

Also the treatment of unilateral hearing loss is possible according to the current guideline; the same criteria apply for hearing loss and speech understanding (in the Freiburg monosyllabic test) as for bilateral hearing loss.

The new guideline provides that the statutory health insurances pay for such hearing devices that are able to compensate the functional deficit of the hearing impairment based on the most recent stage of medical engineering. If possible, speech understanding even with background noise and larger groups of people should be achieved. This decision is based on a decision of the Federal Social Court of Germany dated December 17, 2009, emphasizing the objective that hearing aids have to ensure in the context of an entitlement of benefit.

Another modification of the guideline came into effect on October 29, 2014 [16]. Since the “new regulations on healthcare” from 2013 have abolished the exclusive right of physicians to prescribe aids, this aspect had to be re-defined also for the prescription of hearing aids. Only for first hearing aid use, still medical prescription is required, in cases of consecutive prescriptions it is only needed if a repeated therapeutic decision is requested. The Federal Joint Committee (Gemeinsamer Bundesausschuss der Krankenkassen) decided on July 17, 2014 that prescriptions issued by physicians are needed for consecutive treatment in the following cases:

- children
- hearing loss close to deafness (WHO-4)
- newly occurring tinnitus

From an ENT-specific point of view as well as from the point of view of associations of affected people, these facts are certainly unsatisfactory because even hearing deteriorations of 5 or 10 dB have to be examined medically and not only be cared for with new hearing aids by hearing care professionals.

Finally, those modifications rather have the purpose – as often observed in healthcare – to limit costs, which generally cannot be in the sense of the patients.

In this context, a recent study was published that evaluates the effect of this “reform” on the quality of care: 859 insured people of a statutory health insurance (HHK) were questioned before the reform and 622 were interviewed afterwards. The percentage of those who had to pay own contributions, decreased by 6% (from 80.6 to 74.1%), however, still 40% of the patients paid a contribution of more than 1000 Euro. No improvement of the subjective hearing quality could be assessed, neither of the individual duration of usage. According to the authors’ opinion, the “reform of the hearing aid provision” did not change the lack of compliance of the patients [17].

### 2.1 Overview of new regulations for hearing aids in Germany

In an extensive educational article, Löhler et al. describe in 2 parts the current and newly developed process of hearing aid prescription in the German healthcare system [18], [19]. While in the first part of the article the new rules are explained, the second part contains a presentation of the extension of speech audiometry in the context of hearing aid fitting. The authors present the possible and scientifically sufficiently evaluated application of the Freiburg monosyllabic test in background noise. For the prescribing ENT specialist, those rules and instruments of quality management are essential because they ensure a clearly higher quality and control of hearing aids and avoid separate care of the hearing care professionals that cannot be accepted in the context of satisfactory care for hearing impaired people.

For patients suffering from recognized noise-induced hearing loss with and without tinnitus who are insured via the professional associations, the hearing aid provision has significantly improved due to a new contract between the German Statutory Accident Insurance (Deutsche Gesetzliche Unfallversicherung, DGUV) and the Federal Guild of Hearing Care Professionals (Bundesinnung der Hörgeräteakustiker, BIHA). It exceeds also the regular services of the statutory health insurances. Furthermore, there are 3 categories while the (expensive) category 3 should be the absolute exception. In this contract, also the individual hearing protection is defined. Details have been described by Michel, Wolf, and Brusis [20].

## 3 Process of hearing aid provision: specialist versus hearing care professional – difficulties regarding the treatment of older people

In comparison to most of the other European countries as well as the USA, there is the particular situation in Germany that hearing aids are (generally) prescribed by ENT specialists, but the concrete fitting is performed by hearing care professionals, in rare cases also in ENT departments (mainly in children). The profession of hearing care professional is learned in an apprenticeship (apprentice – assistant – foreman) with an excellent education regarding technique and hearing aid setting, however, completely without therapeutic aspects or authorization. In other countries, hearing aids are either issued and adapted directly by ENT specialists or – even more frequently – by audiologists, which means professionals who are specially trained for the therapy of hearing impaired patients. An advantage of the German proceeding is certainly the high competence in technical equipment and the – at least theoretical – usage of the whole multitude of products even if they are increasingly limited by mechanisms of concentration and market control finally even of the hearing aid manufacturers themselves. In particular forced by the professional association of ENT specialists and accompanied with special courses and additional trainings, the above-mentioned quality control systems are thus very important to control and to verify the provision of hearing aids. Efforts of hearing care professionals to present themselves for example by issuing own “guidelines” as competent also regarding the treatment of hearing impaired and tinnitus affected people, are certainly a wrong and inacceptable way.

With regard to the still missing and insufficient care for hearing impaired people, associated with poor acceptance, social stigmatization, and often too high expectations in hearing aids, it is only possible to improve the situation of the increasing number of hearing impaired people by competent and quality-oriented efforts of ENT specialists, of course in constructive cooperation with hearing care professionals.

In other countries, however, the possibility of hearing aid provision via internet is already investigated. A study from New Zealand enrolled 18 people who were all experienced hearing aid users. They were cared for in this way and then interviewed [21]. The only positive aspect was the reduction of costs and the more comfortable way of purchasing the device. The negative aspects were the poor control and in particular the missing information and consultation by experienced ENT specialists or audiologists.

### 3.1 Side effects of using hearing aids: sweating and development of cerumen

Often sweating causes problems for hearing aid users. Skin reactions may develop, the devices may fail because of increased sweat production; in summary, the wearing comfort is impaired. This is also a reason why hearing aids are often not used during sports. A possible solution regarding those side effects of hearing aid usage is reported from Göttingen [22]: Two cases are presented in which the increased sweat production could be well reduced by intracutaneous injection of botulinum toxin into the temporal skin leading to the effect that the hearing aid could be used again. However, this procedure can certainly only be applied for really excessive sweat production.

Astonishingly, a retrospective study including 164 patients of several audiological departments from England and India came to the conclusion that there is no correlation between cerumen production and density in hearing aid users compared to patients without hearing aids [23].

### 3.2 Difficulties regarding the care of older hearing impaired people

The data situation on the assessment of hearing aid usage especially in older hearing impaired people is rather poor, as confirmed by a meta-analysis from England. 1933 studies were found of which only 64 were selected and evaluated. Only 5 articles were considered as having a sufficient quality, a total of 15 different assessment methods were found for the usage of hearing aids. The authors urgently request better methods for data collection regarding the use of hearing aids [24].

A survey of 1,000 hearing impaired patients in Harvard revealed that only 22% of them used hearing aids. Patients who could achieve an improved speech understanding with hearing aids, had a device in 50% of the cases. Only 0.3% of the patients who did not achieve better understanding used hearing aids. The authors postulate that the word understanding is the crucial argument for purchasing hearing aids [25]. 

It is noteworthy that standardized instruments for assessment of hearing aid use and satisfaction are missing. So each study group develops its own procedure in all different parts of the world, really reliable investigations revealing high evidence are rare.

New epidemiological data still show a poor acceptance of hearing aids, in particular for older people. In the USA, data were compared from 2005 to 2006 with those from 2009 to 2010. A representative sample of 1,636 hearing impaired people older than 70 years were interviewed regarding the provision of hearing aids and the actual usage. Only one third of the hearing aid candidates regularly used the device. In the group that was evaluated later (2009–2010) this percentage was higher. A higher income was related to an increased number of patients who actually used their hearing aids. Regarding gender and further age differentiation, no significant differences were found [26].

In a population-related prospective evaluation from 1993 to 2005 [27], 718 participants with an average age of 70.5 years and a defined hearing loss of more than 25 dB at 0.5, 1, 2, and 4 kHz in the better hearing ear were examined 10 years later. Only 35.7% had received hearing aids. An influence of the educational level of the patients and the recognition of the own hearing loss could be revealed.

A comparable study from Australia [28], also population-related, interviewed 2,956 people with an average age of 67.4 years. A specific hearing questionnaire was used, audiometric data were assessed (PTA). 33% of this group had a significant hearing loss but only 11% wore hearing aids. Among those, 24% never used their device. With increasing age and hearing loss, however, the readiness to use the hearing aid increased.

Hereby, older bilaterally hearing impaired people often used only one hearing aid, even if the binaural provision should be the general rule. As a consequence of this unilateral deprivation, the ability of dichotic hearing significantly decreased. 30 older users of hearing aids (60–81 years) who used only 1 hearing aid, mostly on the right side, could not fulfill the dichotic test items at 100%. 94% only used one hearing aid because 2 devices were perceived as too loud and disturbing, 6% had esthetical reasons [29]. However, hearing aids help affected people also to cope with tasks of their daily life which was shown by an investigation from Brazil showed in 17 first hearing aid users. After 6 months, their activities in daily life improved significantly, especially regarding the use of the telephone [30].

Another study interviewed and examined 30 older and socially active persons on the same topic. 63% had an audiometric hearing loss but only 32% were aware of it. 93% found that hearing aids were too expensive. The authors conclude that socially active older people are frequently aware of their hearing loss but do not want to use hearing aids [31].

In England, 1,874 hearing aid users were asked to fill out a questionnaire; 29% did not regularly wear the hearing aid, especially first users tended to a rather temporary use of their hearing device. Partly only one hearing aid was used despite binaural provision [32].

5,172 older hearing impaired people were evaluated in an investigation from Iceland; only 23% of the male and 15.9% of the female patients used hearing aids. One positive aspect was observed that when people admitted to have a hearing loss, they usually used hearing aids. Women more frequently used hearing aids when they were physically and communicatively more active [33].

The age reduces the hearing improvement in speech understanding. Among 188 patients, the understanding of speech and monosyllables was clearly poorer in 20% of the group of the older people [34].

In this context, directional microphones may help in particular older hearing aid users (n=15) to improve sentence recognition in noise. An efficient suppression of background noise, however, is not sufficiently achieved, as confirmed by a study from Texas [35].

The significance of the age in hearing aid provision was evaluated in 59 patients who had digital hearing aids because of their hearing loss. Hereby, the speech intelligibility and the discrimination were improved in the 65 to 80 year-old people as well as in patients older than 80 years [36].

An interesting article from Toronto evaluated the ability of older patients to operate a hearing device considering especially their dexterity. 20 patients of each age group (18–25 years old, 60–70 years old, and 71–80 years old) were asked to operate a BTE hearing aid. Additionally 28 older patients suffering from arthritis and 28 older patients without joint problems were compared regarding the handling of different controls of the hearing aid. In the first comparison, relevant differences were observed in the age groups but also the test persons with arthritis managed rather well the second experiment and their basic performance regarding the handling was not poorer compared to patients without arthritis. In summary, also older patients were able to operate the hearing aid adequately after training [37].

In a study from Norway, 174 patients of a waiting list of 193 persons for hearing aid provision were asked about their expectancies regarding the hearing aid [38]. The patients were all older than 65 years. They had been referred to hearing aid fitting by their general practitioner. The positive expectancies in moderate to high-grade hearing loss were higher and patients with experience in the use of hearing aids also had higher expectancies. The inhibitions to use a hearing aid were significantly higher in women. Patients with low-grade hearing loss had only low expectancies and wanted to achieve problem-oriented improvements. According to the authors, these facts could explain why patients with low-grade hearing loss only rarely use hearing aids, especially when they do not suffer from disturbing ear noise.

An investigation from Switzerland evaluated 4,979 male and 3,410 female hearing aid users in order to find out how and how long they used their hearing aids per day [39]. Women used their hearing aid clearly more regularly and also for a longer time during the day. In both groups, the duration of using the device decreased when the hearing comfort was low or the device was difficult to operate. The authors request significantly improved fitting and a very intensive support especially in patients with steep threshold reduction in order to achieve the best possible hearing improvement and thus to improve the hearing comfort and the use of the hearing aid.

In Germany, only about 10–15% of the people who actually would need hearing aids (percentage taken from the report of the Green Cross [40]) are provided with hearing aids. Those percentages refer to the guideline of the German ENT Society from 1998, which is no longer up-to-date, on the indication of hearing aids [41]. Comparably poor is the provision of the patients who are older than 60 years. In more than 60% of the participants in an age-related hearing study, an indication for hearing aid rehabilitation was made but only 15% had actually received a hearing device [42]. It was astonishing that many older people did not get any information, neither they had experiences. The percentage of women using hearing aids was significantly higher.

One reason for this poor situation regarding hearing aids for compensation of hearing loss [43] might be social and individual aspects (vanity, stigmatization). On the other hand, according to recent knowledge with regard to deficits in the further hearing processing such as the deficient suppression of background noise, especially the insufficient fitting and quality of hearing aids and the associated patient compliance play a decisive – negative – role. 

### Conclusion

Especially because hearing in noise is most severely impaired [44], an optimal suppression of background noise [45] should have the absolute priority for hearing impaired people with high central deficits – even if it is at the expense of optimal sound quality. In this context, it is astonishing that increasingly more modern hearing aids are developed without induction coil (perhaps because of the size), because in particular for optimal suppression of background noise, induction coils used in meetings, in church, or concerts are most suitable and a great help for older people. However, still too few meeting locations are accordingly equipped – the association of hearing impaired people often claims improvements that are only rarely implemented [46].

## 4 Diagnostics before hearing aid provision

ENT-specific diagnostics are obligatory in Germany at least before first fitting of hearing aids. In general, it ends up with the above-mentioned indication for hearing aid provision and the prescription of one or two hearing aids. The main objectives of hearing diagnostics are the determination of the severity, i.e. the quantification of an existing hearing loss, and the identification of the origin, i.e. the localization of the hearing impairment.

### 4.1 Subjective audiometry

The most important examination methods in this context are tone and speech audiometry measured with the Freiburg monosyllabic and number test. For both aspects, the measurement results have to be noted in a form issued by the statutory healthcare services. In this way, the type of damage (sensory hearing loss or conductive hearing loss) and the severity of the hearing impairment can be directly identified as frequency-related hearing threshold. The statutory health insurances refer to the above-mentioned indication criteria regarding tone and speech audiogram. Exceptions – for example low-grade hearing impairments – require extensive explanations and justifications.

### 4.2 Objective audiometry

To confirm and objectify subjective audiometry, objective measurements such as impedance audiometry (tympanometry and stapedius reflexes), the measurement of otoacoustic emissions (OAE) and the electric response audiometry (brainstem electric response audiometry, BERA; cortical electric response audiometry, CERA) may be applied. They are highly relevant for pediatric audiological hearing diagnostics, but also in the context of aggravation, simulation, and dissimilation and in patients who do not sufficiently speak the (German) language. 

Objective audiometric measurements are furthermore important tools for the localization of hearing disorders. So they should always be performed in cases of suspected retrocochlear damage or central lesions. Another differential diagnostic questions concerns the confirmation of auditory synaptopathy/auditory neuropathy (auditory neuropathy spectrum disorders, ANSD). Hereby, regular OAE may be measured even in cases of high-grade hearing loss while the brainstem potentials in BERA completely miss or are suspect. The prevalence was estimated as rather low in the past, but recent evaluations especially of high-grade hearing losses in children confirm case numbers in a range of some percent [47].

The quantification of the hearing impairment can be assessed by questionnaire inventories. The severity of the impairment by hearing loss gives first hints to the later usage of hearing aids and can thus be applied to answer the question if the patient is ready to use the device as it is explicitly required by the guideline on hearing aids. 

Beyond basic care, the provision of hearing aids for minimal hearing losses is increasingly discussed [48], [49]. Due to the improved technology of hearing aids, the devices can contribute efficiently to direction-selective suppression of background noise beside sound amplification and thus improve the signal-noise distance in certain everyday situations.

## 5 Historical aspects of device-related hearing rehabilitation

Already a hand held behind the ear amplifies sound signals in the frequency range of 1–2 kHz and 6–8 kHz of up to 16 dB [50]. Beside this simplest method, in the pre-electronic times ear trumpets – for example animal horns – were used. The amplifying effect of those trumpets was mainly based on the reduction of the surface from the opening of the sound entrance to the opening of the sound exit. With ear trumpets, the amplification amounts to 20–30 dB, the main amplification, however, is in the frequency range below 2 kHz [50].

The development of hearing aid technology was always coupled to the general development of electronic components. In the first decades of the 20^th^ century, the first pocket hearing aids were developed on the basis of electronic tube amplifiers. The implementation of transistor technology in the 1950ies and later of the integrated circuits (IC) rapidly led to further improvement of the amplificatory performance and the size reduction of the devices [51].

The first hearing aids that were fixed behind the ear (BTE) were applied already in the 1950ies, the first devices in the ear (ITE) were used in the 1960ies. An important technological development was the possibility of adjusting the tools via a computer (digital programming). In this way, reproducible settings became possible and the base was found for a parameter-based and reproducible setting. In the mid-nineties of the last century (in 1996), the first completely digital hearing aid came into the market. Initially, the digital technology led to irritations in some hearing aid users because these expensive tools were promoted intensively without being more effective. Especially people with high-grade hearing losses did not benefit at all from those devices. After extensive hardware and software optimizations, the digital devices became much better and could completely replace the analogue hearing aids.

The device-related hearing rehabilitation was always associated with stigmatization. This fact is confirmed by the above-mentioned usage data of hearing aids. The limited hearing ability can be hidden by discreet compensation strategies up to a certain level even if many everyday sounds are not heard. Even if much has been changed over the years, the invisibility of a hearing prosthesis is still an important concern of the users. Often it still impairs the quality of adequate care.

## 6 Technical basics of hearing aids

The basic effect of hearing aids is the amplification of the sound. It has to be adapted to the hearing loss identified in the tone audiogram depending on the frequencies. Furthermore, the amplification has to correlate with the input level. For lower levels generally a higher amplification should be set than for higher levels. Finally, a series of additional signal processing measures is performed in order to allow better hearing in specific hearing situations.

### 6.1 Functional principle

**Figure 2:**
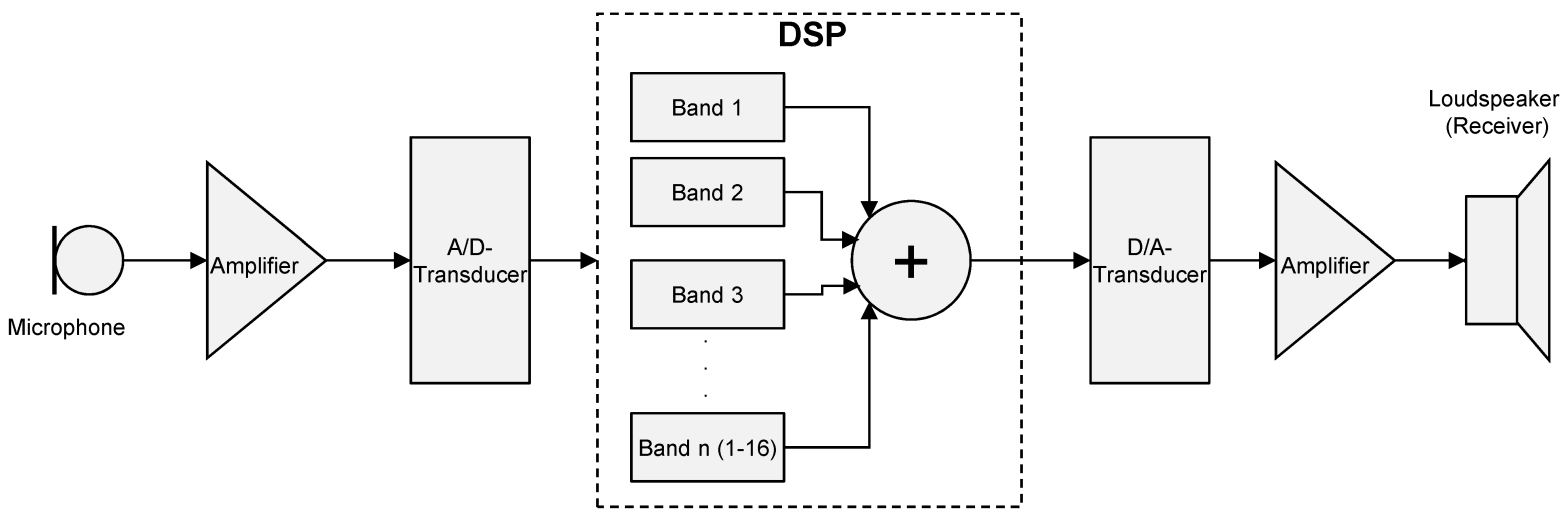
Functional principle of modern digital hearing devices

Modern hearing aids are based on a completely digital signal processing. The sound is received by a microphone and transformed into an electric signal and by an analogue-digital transducer into a discreet pulse sequence. Typically, the sampling rates of modern hearing aids amount to around 20 kHz (compared to 44.1 kHz in CD players). In this way, already a theoretical limit of 10 kHz for further signal representation and processing is determined. The discrete signals are separated in the hearing device into several (4–20 channels) frequency ranges. For each channel, the signal processing is separately performed including the frequency-specific amplification. Then, the single signals are merges, amplified and after digital-analogue transduction the sound is emitted via a miniature loudspeaker (Figure 2 (Fig. 2)).

The relevant signal processing occurs according to the channel in a signal processor (DSP). The number of channels limits the number of independently adjustable frequency ranges. This number for frequency-depending hearing losses plays a central role. Since typically only 10 different frequency values are tested in tone audiometry, the number of channels is no longer a challenge from a technical point of view.

#### 6.1.1 Types of amplification

**Figure 3:**
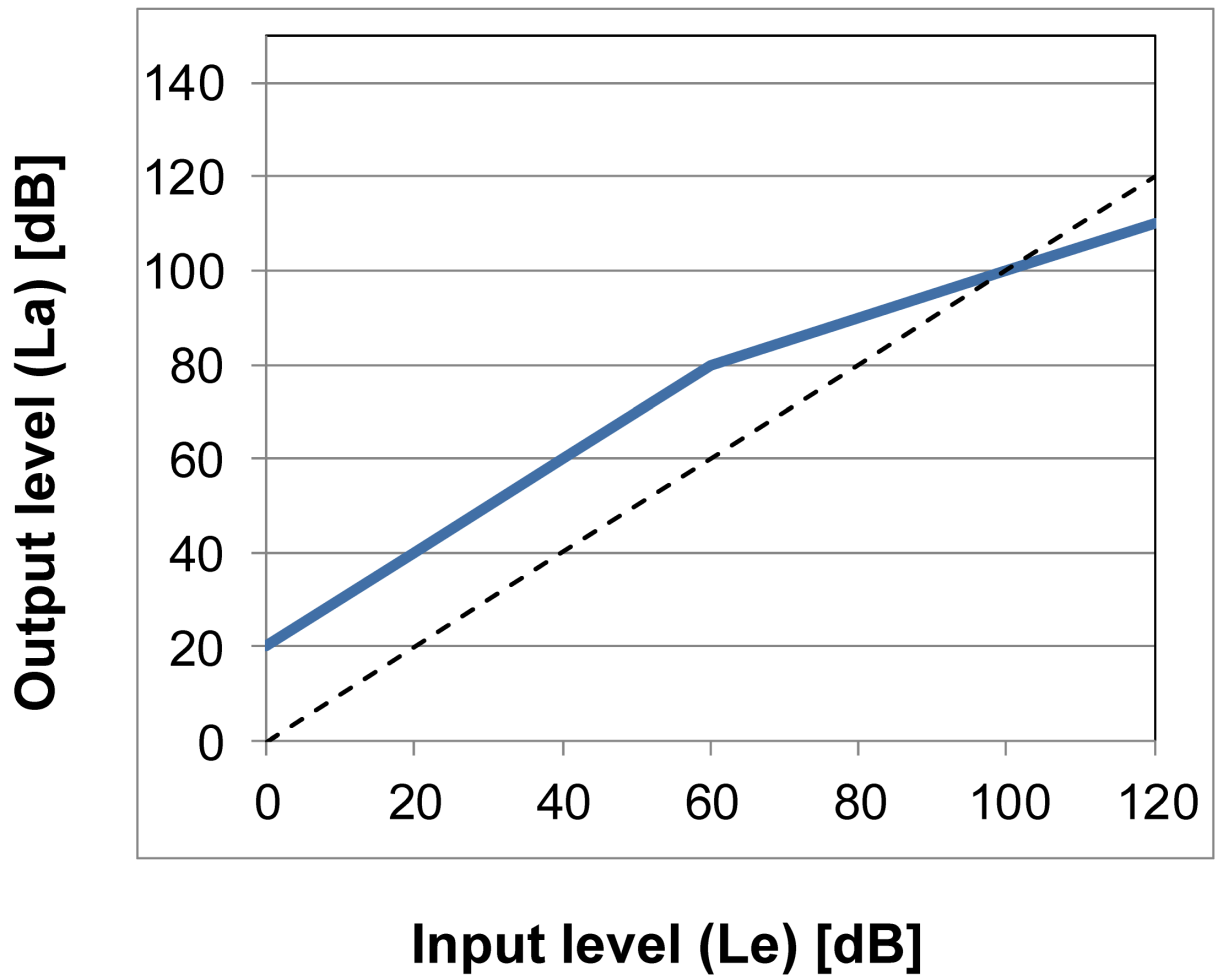
Output level of a hearing aid as curve of the input level with non-linear amplification (thick line) in comparison to the unamplified signal (dotted line). The amplification below the knee-point is 20 dB, above the knee-point of 60 dB it decreases linearly. Above 100 dB, even signal attenuation occurs. The whole amplification process may be described by the initial amplification, the knee-point, and the compression.

The amplification must be adapted to the type and severity of the hearing loss. For this purpose, linear and non-linear amplifications are available. In the context of linear amplification, all input levels are amplified to the same extent. In the level range, this corresponds to an increase of a constant decibel value. This type of amplification is suitable for low-grade sensory hearing losses or conductive hearing losses. In cases of higher-grade hearing losses with defined recruitment a lower amplification has to be set for higher input levels in order to take into consideration the discomfort limit. This is achieved by compression. Typically, a linear amplification is applied up to a certain input level, for higher levels it is then reduced. Figure 3 (Fig. 3) depicts a scheme of this correlation. The non-linearity can be described by the knee point and the compression ratio. Furthermore, there are other compression variants that function in a similar way.

Another special, non-linear type of amplification is achieved by frequency reduction [52]. It is realized for example as frequency transposition or frequency compression. Hereby the spectral properties of the sound signals are modified. The background of this frequency reduction is that most hearing losses are high-frequency related and often an amplification in the high-frequencies is no longer useful. In those cases, the frequency reduction of the sound signals leads to hearing. These procedures are associated with unavoidable signal distortions. In many cases, however, a better hearing perception can be achieved after some months of acclimatization. Since such frequency reduction procedures are initially disturbing, the actually adjustable realizations in commercially available hearing aids are rather reluctant. They do not lead primarily to a better speech understanding but mainly improve the sound impression [53]. Up to now, there is no possibility to identify particular subjects who might benefit from frequency reduction.

In contrast to earlier analogue hearing aids, not only the hardware but also the quality of technical components in digital hearing devices is essential. The function of a hearing aid is determined by the signal processing defined in the software. Thus, there are today technically identical hearing aids that are only different regarding their technical features (and price). The software determines for example the feedback suppression, wind noise suppression, directional microphones etc. (see below).

### 6.2 Construction types of hearing aids

Hearing aids are classified according to different criteria. In the practice, there are always different quality levels (from “standard” to “premium”). This commercial classification primarily refers to the selling price, secondarily to the connection to phones etc. and only then to the actual signal quality. Because of the healthcare situation in Germany, nearly all hearing aid types have a standard device that is sold at the currently valid sum.

Another criterion refers to the wearing variant of the hearing aid. Nowadays most devices are fixed behind the ear (BTE). The sound is registered by the hearing device above the auricle, processed, and conducted into the auditory canal either electrically or via sound tube. In cases of electric transmission, the sound is finally applied via a miniature loudspeaker that is located in the auditory canal (ex-hearer or receiver in the canal, RIC). Only about 10% of the available hearing aids are used in the ear (ITE). Here, the difference is made between those that are located completely in the canal (CIC) and those that are completely or partly located in the concha (concha devices) [54]. From an audiological point of view, such devices do only have few advantages and are often disadvantageous because cosmetic parameters are preferred to technological possibilities. The advantage that is obtained by the acoustic effect of the auricle is clearly lower than the disadvantage of the simple technique. Another variant are hearing aid glasses. Hereby the hearing aid is completely integrated in the frame of the glasses where a nearly invisible cable leads to a loudspeaker located in the ear canal. This option reduces the number of necessary devices in the context of vision problems and hearing loss but in cases of defects and repairing both senses are impaired.

Another differentiation of the device-related hearing care is defined by the degree of sealing of the auditory canal. If an acoustic bridge exists between the microphone and the receiver of the hearing aid, the output signal is again amplified and a technical derailment of the amplification occurs with the typical peeping of the hearing aid. In the past, this failure known as feedback could only be avoided by occlusion of the auditory canal. Thus, nearly all fittings were performed in a closed way prior to the introduction of digital hearing aids. The effect was the minimization of the correlation between amplified sound and non-amplified sound. The necessary, individually manufactured earmolds also lead to the fact that only the sound is perceived that is amplified by the hearing aid and the occlusion of the auditory canal leads to increased own body sound (occlusion effect). In order to reduce this phenomenon, vents are made of 0.5 to some millimeters that lead to an improved sound. In even more extreme cases, earmolds can be avoided and only the sound tube or the receiver are placed in the auditory canal without occlusion. This type of open fitting is suitable for hearing losses that require low amplification – especially in the frequency range below 1 kHz.

All mentioned hearing aids are not intended to be used for 24 hours. In order to allow this, a device was developed that is located completely in the auditory canal (“hearing lens”) that may remain in the auditory meatus for several months. Due to the very close location directly at the eardrum, the power can be supplied by only one battery for some weeks. However, since this battery is integrated in the device, the apparatus has to be regularly changed after 8–12 weeks. The change of the aid should be performed by experts under microscopic view. The advantage of such hearing aids is the use during the whole day and at nighttime, the invisibility, and the absence of wind noise. The disadvantages are high expenses and the limitation to only low-grade to max. moderate hearing losses.

Hearing aids are also different with regard to the amplification performance. Even if theoretically a broad spectrum of settings is possible for the amplification of a hearing aid, differentiations must be made in the practical use. The expectation concerning hearing aids for low-grade hearing losses is mainly the preservation of a natural sound quality with low amplification. For this purpose, the inherent noise caused by the amplifier has to be minimized and the sound signal has to be preserved broadband. The user is especially interested in the improvement of the speech intelligibility in complex hearing situations with background noise and the better hearing of music. In the context of higher-grade hearing losses, in particular the sufficient amplification performance is important to achieve better speech understanding in quiet (Power, High power, Super power). Beside the upper level limit, also the suppression of feedback is relevant. To take into account “dead regions”, the amplification of the high-frequency range is often waived or as described above, frequency reduction may be tried.

## 7 Modern hearing aids: modifications and improvements

Modern hearing aids are miniaturized high-power computers that have to function with little energy for a possibly long duration. While formerly hearing aids were limited by the components (microphone, amplifier, loudspeaker), the quality of a modern hearing device is rather defined by the immanent software. Even if there are permanent improvements of the hearing aid technology, exaggerated advertising measures often lead to disappointed users because the effectiveness of the single improvements is not always noticeable. Nonetheless, several improvements could be observed in the past years.

### 7.1 Directional microphones

Directional microphones are useful when the speech signal comes from the front and the background noise from other directions. Nowadays, directional microphones cannot only be implemented as fixed part but they can also be digitally added. Adaptive directional microphones only switch on when a speech signal is recognized as coming from the front. This is possible due to the evaluation of the time delay of the sound input at 2 different microphones.

### 7.2 Wind noise elimination

The wind that reaches the open ear without hearing aid is mostly well eliminated by healthy hearers and only rarely leads to noticeable hearing deterioration. Since most of the hearing aid microphones are located above the auricle, the natural protection is missing. Furthermore, most microphones have a directional effect set to the front. Thus, also low wind velocities lead to significant deterioration of the signal quality. In the past, this fact led to dissatisfaction in nearly half of the hearing aid users [55]. A series of procedures for reduction of wind noise was developed. They aim mainly at increasing the wearing comfort and in second place also speech understanding in those situations is improved.

### 7.3 Binaural coupling

Coupling two hearing aids of one user became possible after the development of an appropriate wireless protocol. The first steps were already made several years ago and led to the presentation of the German Future Award in 2012. Initially, binaural coupling was limited to the (wireless) transmission of parameter settings of one hearing aid to another. In this context, for example, the change of the loudness in one hearing aid was also effective in the other one. Later, this feature was extended by real time transmission of audiodata. In this way, a very important directional effect of the microphones can be achieved. Additionally, the hearing aid can recognize on which side the speech signal is presented and amplify it. During phone calls, such devices can transmit the telephone signal to both ears. Even if some of the hearing aids are already in the market, the usage of coupling two hearing aids will certainly be optimized in the next years. It is highly efficient to use binaural coupling also for elimination of wind noise when they only occur in one ear.

The current hearing aids with binaural coupling are still far from providing real binaural processing. Here, an enormous development potential exists.

### 7.4 Future developments

Most recent development indicates a tendency that also entertainment companies will enter the hearing aid market. A hearing aid of the future could then possibly be a headset worn at the ear that wirelessly sends data to a smartphone, receives the processed signal and sends it back to the ear. The complex signal processing can be performed by the smartphone. The function of the hearing aid would then be limited to sound reception and sending. The coupling to the phone would then be inherent and other devices could be connected via Bluetooth. In some areas, such a hearing device might be sufficient and also be reasonable as entry-level device. However, it requires a high level of knowledge, self-control and can certainly not be applied in any patient group and higher-grade hearing losses.

## 8 Additional equipment

Hearing aid manufacturers provide a series of additional equipment for their hearing systems that might be useful in individual cases. Those are wireless remote controls of the hearing aids that are for example integrated in a watch and that can switch discretely between different hearing programs.

Another additional equipment concerns coupling to other acoustic systems such as telephone, TV set, or music players (MP3 players). Phone calls are a great problem for many hearing aid users. On the one hand, the additional visual information is missing for communication. On the other hand, coupling of the acoustic signal from the telephone receiver to the hearing aid microphone is generally not stable. In this context, already several years ago the telephone coil was integrated in the hearing aids. Since former telephone receivers generated a sufficiently alternating electromagnetic field, sound could be received by an induction coil (T coil) implemented in the hearing aid. The hearing aid had to be switched to a specific program. But since the electromagnetic field strength of modern telephones is no longer sufficient, it is no longer possible with all telephones. The T coil, however, can also be used for the reception of induction loops integrated in the floor. This possibility of hearing via a T coil is the standard today in many churches, theatres, music and lecture halls. It is a comparably cheap wireless transmission and furthermore effectively suppresses background noise.

Individual wireless transmission devices are still often based on analogue or digital frequency modulation technique (FM). In comparison to induction transmission, the signal quality is better and it is also possible over longer distances. It is appropriate when understanding a speaker is particularly relevant for the hearing aid user. Thus, FM systems are often used in schools. FM transmission can be prescribed according to the guidelines on aids prescriptions. They can be prescribed as soon as they are necessary to meet the basic needs of daily life. This concerns for example children at school age. Regarding adults, it has to be checked carefully and individually if an FM system is necessary to meet general needs of daily life.

In the context of wireless coupling of telephone, TV etc. to hearing aids, different solutions have been presented in the past years. Recently, the first hearing aid that can be coupled to a smartphone was presented (“iPhone hearing aid”). The standard of Bluetooth that is applied in many fields of audio communication is generally applicable but it still needs disproportionately much energy. The further development of this technology will probably lead to a complete transformation of the hearing aid market.

### 8.1 Signaling equipment

In cases of severe hearing loss, an acoustic alarm is no longer possible. For this purpose, a series of additional equipment exists that functions via vibration or flash signals. Light signal systems, vibration messages, light and vibration alarms can generally be prescribed within the regulations of the statutory health insurances since they belong to the group of communication aids. In the context of signal provision, there are also systems solutions that can be connected to different signal generators such as door bell, telephone ringing, smoke alarms, or baby-phones. 

## 9 Fitting process of hearing aids

The legal framework of hearing aid provision in Germany is built by the social law, put in concrete terms by the guidelines on hearing aid prescription and finally also implemented in contracts with the cost bearers. The role of the ENT specialist was increasingly restricted during the last years. Today, medical prescriptions for hearing aids are only obligatory for first users, in cases of hearing deterioration, and for children. The formerly obligatory medical confirmation of successful hearing aid fitting has already been abolished by some health insurances. Nonetheless, the ENT specialist is the audiological contact person for most hearing impaired patients and thus he should be informed intensively about the fitting process.

The fitting of hearing aids is a process of several weeks and months in which the most suitable hearing aid is identified and the optimal settings for daily life are set [56].

First, the audiological profile is determined by measuring the severity and the type of hearing loss and the individual needs are assessed. It is clarified which hearing situations appear frequently and are especially relevant. How important is the improvement of speech understanding in quiet compared to understanding in noise? How important is coupling of a hearing aid to a phone? Are there frequently lecture situations (e.g. school children or students), are there specific directional constellations (e.g. taxi drivers), or are there special requirements regarding dirt and water repellency (e.g. pool attendants)? Are highly different settings constellations necessary? Does switching have to be automatized or at the touch of a button? Those and other similar questions lead to a selection of the possible hearing aids.

The initial setting of the hearing aid is generally performed software-assisted on the base of audiometric parameters such as hearing thresholds (air and bone conduction), if needed completed by discomfort thresholds or data of the loudness scaling. Because of the inexactness of the measurements, most hearing aid fittings are performed only on the basis of a tone audiogram. The discomfort thresholds are estimated by the settings programs.

The objective of compensation in cases of sensory hearing losses is never the complete amplification of an existing hearing loss. The target amplification is rather at half of the hearing loss. Most fitting formulas such as NAL (National Acoustics Laboratories of Australia [57]), POGO (Prescription of Gain and Output [58]) generally correct the values that are adjusted downwards for higher frequencies and upwards for lower frequencies. Other procedures such as DSL (desired sensation level [59]) try to restore the loudness sensation via the dynamic range and thus require the individual loudness scale over several frequencies.

In the practice, hearing aid fitting is completely performed by the software provided by the hearing aid manufacturer. Based on the audiometric data, the prescription is calculated and the hearing aid is programmed. Additional information such as unexperienced or experienced hearing aid user lead to further automatic adjustments.

Furthermore, other modifications of the hearing aid settings can be performed after testing the device in daily routine. This fine tuning then takes into account the hearing environment and also the sound quality may be optimized.

### 9.1 Comparative fitting

For hearing aid provision, attempts should be made with different types of hearing aids. Only this paired comparison will give the users the possibility to assess their quality. The devices should be tried out at home over an appropriate period of time and with every type at least one speech audiometric measurement should be performed for comparison.

Most cost bearers obligatorily include probatory fitting of at least 3 different devices and require the provision of the hearing aid with the best speech audiometric result.

### 9.2 Flexible fitting

In most of the cases, initially the actually necessary amplification is not tolerated. The transition to the changed sound is perceived as strange so that hearing habituation is needed. This may take up to several weeks or months. Especially in cases where hearing deprivation was present for many years, further adjustments are essential. For this purpose, hearing aid manufacturers provide so-called acclimatization grades from “beginner” to “experienced hearing aid user” as option in the software and performed an according modification of the hearing aid fitting.

After some time, the amplification may then be increased to the actually necessary level. Some hearing aids are able to automatically perform a slow increase, while the hearing care professional has to define the velocity of upregulation. Such procedures have the advantage that they avoid frequent appointments with the hearing care professionals but they may also lead to a refusal of the hearing aid that is not noticed by the hearing care professional.

## 10 Quality control of fitting

In contrast to visual aids, the success of hearing aids is only perceived to a limited extent. Typically, users describe initially that sound is better heard but that speech understanding is not improved. Often the new sound is perceived as uncomfortable. Furthermore, hearing improvement is not given in all situations of everyday life. So the quality of fitting can only be assessed with additional measurements. The evaluation of the success of hearing aid fitting is based on three pillars:

- technical verification of the hearing aid fitting
- audiometric measurements with and without hearing aid
- assessment of everyday hearing by the user based on questionnaire inventories (validation)

### 10.1 Technical verification

The technical verification of the measurements is used to confirm that the hearing aid works in a way that is appropriate for the individual hearing loss (verification). This is performed by comparing the sound reaching the microphone with the one in the auditory canal. Hereby the auditory canal may be stimulated by a 2 cm^3^ coupler (coupler measurement) or a sound probe is directly placed in the auditory canal and then the sound pressure directly in front of the concha is compared to the one measured via the sound probe (in situ measurement).

Since modern hearing aids dispose of a series of intelligent noise elimination procedures, the technical verification with sinus tones or narrowband noise has only limited value. They are possibly recognized as interfering signals and eliminated by the hearing aid. More appropriate is the use of defined speech signals or pseudo speech signals [60]. By means of a percentile analysis, a frequency-related graph is created showing the percentage of speech in the hearable range. This type of hearing aid verification is an important component in particular in hearing aid fitting of children.

### 10.2 Audiometry

Tone audiometric measurements in the free-field with wobble tones or narrowband noise with and without hearing aids allow an estimation of the frequency-related amplification. Loudness scaling may be performed to assess the supra-threshold behavior of hearing aids. With speech audiometric measurements in quiet and in noise, the individual improvement of speech understanding is confirmed.

The speech audiometric tests have to be adapted to the vocabulary. So especially for children, adapted test procedures have to be chosen [61]. In adults, the Freiburg monosyllabic test is suitable for measurements in quiet [62].

**Figure 4:**
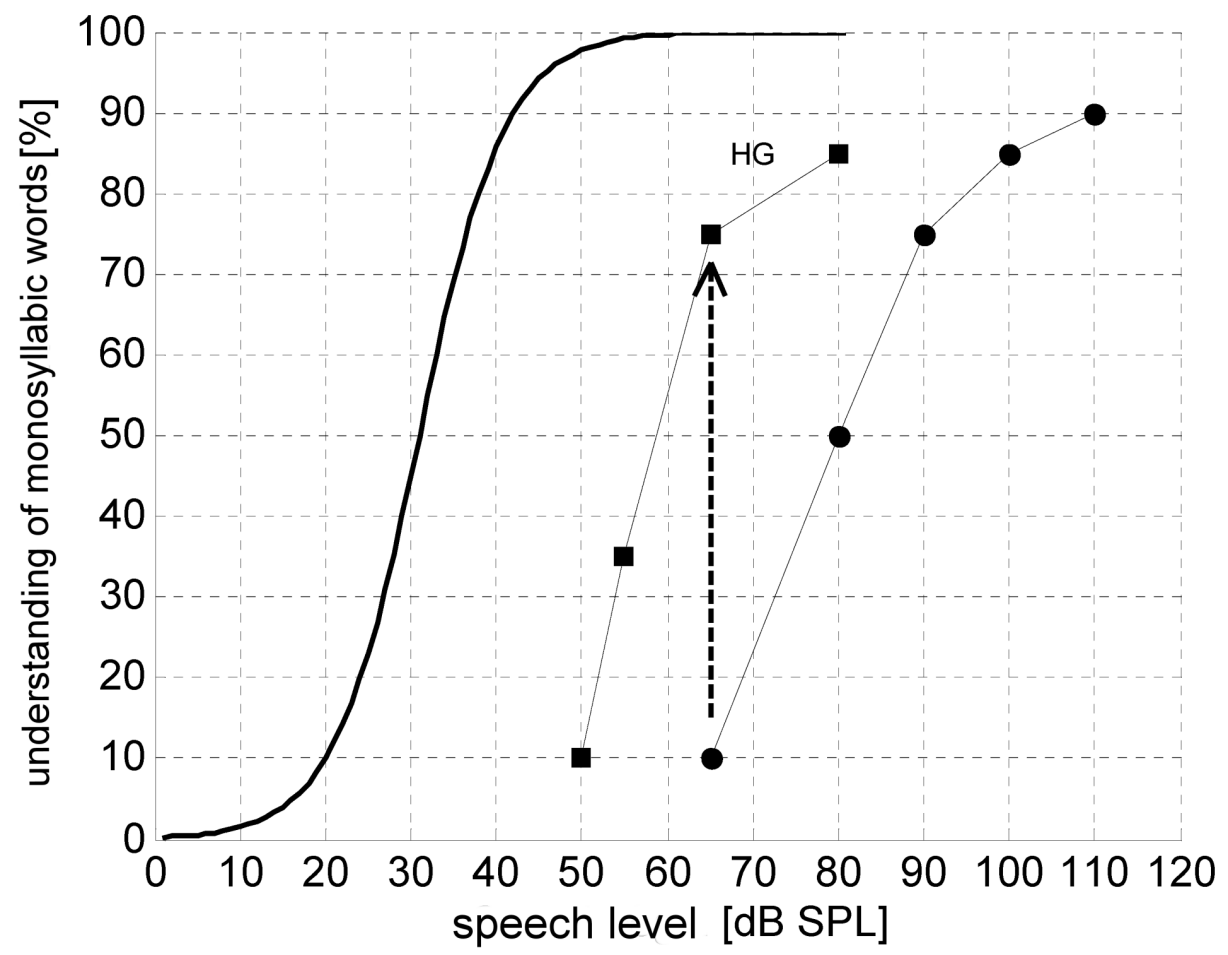
Example for typical hearing aid care. The figure shows the understanding of monosyllabic words without (circles) and with hearing aid (rectangles) in dependence from the speech level. For everyday speech, the speech intelligibility was improved from 10 to 75%. The average understanding of monosyllables of 90% is only merely achieved with hearing aids. The standard curve for normally hearing people is significantly better. In the level area, the discrimination curve is shifted of 20–25 dB to the lower values.

The Freiburg monosyllabic test is used in speech audiometry which is an obligatory part of hearing aid prescription. Thus, understanding of monosyllables can be used as objective for hearing aid provision. The maximally achieved understanding of monosyllables in the speech audiogram should nearly be reached at 65 dB with hearing aid and not decrease with higher sound levels. Figure 4 (Fig. 4) shows an example. In practice, the average values that can be achieved with hearing aids are 10–20% below the maximal understanding of monosyllables because the necessary amplification is not always tolerated [63]. This is especially the case for older hearing aid users or when the discomfort threshold is very close to the level of optimal understanding [34].

The speech audiometric control in noise plays an increasing role because modern hearing aids always better eliminate background noise. However, it is more difficult to standardize speech audiometry in noise. Beside the type (syllables, monosyllables, polysyllables, sentences) and the level of the speech material, the result of a measurement is influenced by several other parameters. In noise, not only the spectral composition plays a role (white noise, speech masking or speech simulation) but also the timely properties (continuous, interrupted, speech modulated). Further, the spatial arrangement of the loudspeakers (speech and noise coming from identical or different loudspeakers) is important. Finally the absolute noise level is relevant and its correlation with the speech level. Since the noise elimination procedures of the hearing aids sensibly depend on those parameters, the speech audiometric control of hearing aids in noise is limited to standard measurements with monosyllables. From a theoretical point of view, sentence tests are more precise such as the Göttingen test or the Oldenburg test. They allow measurements of the speech intelligibility threshold in noise. A speech intelligibility threshold of 50 means a signal-to-noise ratio (SNR) where 50% of the presented speech material are correctly repeated. The measurement is usually performed in an adaptive way. This means that the speech level remains constant at for example 65 dB. Depending on the number of correctly repeated words, the noise level is automatically varied until about half of the words are understood. The advantage of this adaptive measurement is that it is performed in the steepest part of the discrimination curve. Even small changes, e.g. by hearing aids, lead to different speech intelligibility thresholds. The disadvantage of those measurements of the speech intelligibility threshold in the context of hearing aid fitting is that hearing under everyday circumstances usually occurs in more favorable situations where clearly more than 50% are understood. So it is possible to have a highly reliable measurement that does not imperatively reflect the quality of everyday hearing and thus does not allow statements on the actual advantage of usage [62].

The current guidelines on the prescription of hearing aids [16] do not mention sentence tests as precondition for the indication. But they provide a measurement variant that should be used for testing the intelligibility in noise: When verifying the results of hearing aid fitting in the free-field by means of the Oldenburg sentence test (OLSA; after elimination of the training effect) or of the Göttingen sentence test (GÖSA), the speech intelligibility threshold measured binaurally in speech simulating noise of 45 dB without hearing aids in the same spatial situation should be reduced of >2dB S/N (signal to noise) of the speech intelligibility threshold.

The mentioned limit value of >2 dB corresponds roughly to the former criterion of improvement of 20% [64]. This kind of measurement should assess the different problems of hearing impaired people with one measurement method. In cases of low-grade hearing loss, the speech understanding in noise is in the focus, in cases of higher-grade hearing losses also the speech understanding in quiet is impaired. By introducing this low noise level that is merely perceived by severely hearing impaired patients, both problems are addressed. The suggested binaural measurement corresponds on one hand more to the real everyday situation. On the other hand, the correlation of the binaural speech intelligibility threshold and the everyday hearing is not yet clarified. So discrepancies between the measurements and the subjectively perceived everyday hearing are observed especially in highly frequency-related hearing losses and asymmetric hearing [63], [65].

### 10.3 Subjective assessment

The most important aspect of hearing aid provision for the individual person is the subjectively experienced benefit. Generic questionnaires to assess the quality of life are often too general and thus not suitable. For this reason, numerous specific questionnaires on hearing-related quality of life, understanding of everyday speech, directional hearing, or perceived sound quality have been developed. The most comprehensive data in the German speaking countries are currently available for the APHAB (abbreviated profile of hearing aid benefit). Internationally, however, also some other inventories such as the SSQ (speech, spatial and qualities of hearing scales) or the IOI-HA (international outcome inventory for hearing aids). Also the Oldenburg inventory is appropriate to assess speech intelligibility in daily life.

### Conclusion

The fitting of hearing aids is a long and complex process. A quality control is obligatory because the affected people cannot assess the benefit themselves. Thus an evaluation of hearing aids is always based on three pillars (technical measurement control, speech audiometry, and subjective assessment). Only then it is possible to evaluate if the hearing aid provision is sufficient, suitable, and appropriate.

## 11 Necessity of early hearing rehabilitation and hearing aid provision as early as possible – what does the benefit depend on?

In modern otolaryngology, the increasing tendency is observed to adequately treat hearing impaired people as early as possible even for milder hearing loss. However, still the acceptance of hearing aids is not very high due to different reasons. Partly there is still the problem of existing or feared stigmatization by the hearing aids, certainly also too high expectations that people have of hearing aids, are a reason. So, hearing aid provision must be associated with significantly improved information and at the same time suitable and realistic consultation. Therefore, validated and reliable instruments are needed to assess the individual benefit.

A first model was developed by a group from the ENT department of Cologne in 2008 as a model to predict the hearing aid acceptance. 100 hearing impaired people without hearing aids were asked to fill out a questionnaire that included beside the readiness to use hearing aids also different socio-demographic data and the expectations regarding hearing aid fitting. The expectations were relatively high and undifferentiated. These questionnaires were the base for a model to predict the later usage of hearing aids [66].

For Germany, in the last years a sophisticated questionnaire to assess hearing aid benefit was developed by Löhler and co-workers supported by the professional association of otolaryngologists and presented in several publications [67], [68]. This APHAB questionnaire (abbreviated profile of hearing aid benefit) meanwhile includes an extensive database of which a series of important knowledge could be generated beside the necessary quality control that will be presented in the following paragraphs.

The APHAB database and the questionnaire were presented in the journal *HNO* [69]: A survey of patients is obligatory for accounting of hearing aids, the APHAB questionnaire is validated for Germany as instrument of quality management. It focuses on different hearing situations. The simple hearing situation, hearing in noise, hearing of speech in echo and reverberation situations, and hearing in loud surroundings. The questionnaire is available in 21 languages and can be applied broadly. It will be a very well evaluable database with easily searchable data in the future. The questionnaire may be retrieved after registration in the internet: http://www.quihz.de.

According to a recent survey with this APHAB questionnaire evaluated for Germany only 70% of hearing aid users report about a benefit. The authors postulate that obligatory ENT-specific control of the hearing aids and good consultation with the hearing care professionals are required [70].

A first evaluation of 2,745 questionnaire revealed that the APHAB is suitable for assessment of hearing impairment, independent from later hearing aid provision [71].

In a more recent evaluation of 23,557 questionnaires, it could be shown that most questions provide reliable quality control statements. In contrast, other questions referring to cinema, theatre, or church visits have been less answered [72].

Additionally, the questionnaire helps especially before hearing aid fitting to understand specific frequency-related intelligibility problems of the patients [73].

So in Germany, we have a well applicable instrument for assessment of hearing impaired people and the control and quality management of hearing aid fitting that should be distributed broadly and applied intensively.

From other countries, single studies were presented that try to collect similar information, however, they are less systematic and thus cannot be assessed and evaluated in the same way. A review from the USA includes articles about hearing aid users with <45 dB HL at 500, 1000, 2000, and 4000 Hz. 106 articles were evaluated, 10 of them were finally selected: with an effect size of 0.85% (95% confidence interval) a high evidence was found for the positive effect of hearing aids for mild hearing loss [74].

Also in the USA, data on the operation of hearing aids and the user satisfaction were evaluated: 47 hearing aid users (first users and experienced ones) were asked to fill out questionnaires (hearing handicap and later satisfaction) on hearing aids. For the majority of the people, the operation was well possible but only few people managed more specific fields of application. The satisfaction was not very high, however, it depended from the costs and the provided service [75].

From Brazil (Sao Paulo) a study with questionnaires and specific tests was published that included 25 older (69.7 years) hearing aid users. The conclusion was that education, cognitive abilities, and possibly present depression significantly influence the performance [76].

A trial from Japan found out that the success of hearing aids depends on the quality of life of the hearing impaired people. 157 patients were included in the study who were older than 65 years. People with higher life satisfaction use their hearing aids more often and more consequently, audiological parameters, however, only have a minor impact [77].

In Australia, 468 hearing aid users and 26 audiologists were asked. The authors found out that the influence of physicians and caring professionals was not relevant for the use of hearing aids. Even a frequent change of the audiologists did not lead to poorer results [78].

From 68 hearing aid users evaluated in an article from Nottingham, 32 received a special motivation support, 36 had standard provision. Initially, there was a higher commitment and readiness in the supported patients but after 10 weeks no more differences were found [79].

45 hearing aid users from the USA extensively tested 4 different types of hearing aids (basic and premium). No significant differences were found regarding satisfaction of both types of hearing aids. The data were evaluated with regard to quality of life, hearing quality in daily life, and the difference between basic and premium devices [80].

An interesting study from Netherland investigated the effect of hearing improvement by the use of hearing aids in terms of emotional factors and parameters. They compared 23 hearing impaired people with and without hearing aids with a control group (n=22). The affective perception, the emotional components, and the interpretation of excitation patterns could be improved with hearing aids [81].

It is also interesting that an intercultural comparison of the hearing aid acceptance in different countries revealed that the social acceptance of hearing aids is different in different cultures. The study compared the specific situations in India, Iran, Portugal, and England [82], [83].

### Conclusion

In summary, the statements about the benefit that hearing impaired people have from hearing aids, are rather contradictory. From the high number of the studies of the last 2 years that were presented here, the evaluations and results show again the necessity to assess the data in a more systematic and exact way. With the APHAB questionnaire applied in Germany, we have an excellent base that will certainly generate new knowledge due to the increasing database. Already now it can be stated that the provision can and must still be significantly optimized, but that also control and follow-up have to be intensified. It is especially important to inform hearing aid users before fitting in order to assess and correct the individual expectations, in particular when they are unrealistic after concrete hearing loss. Perhaps, also the technology of hearing aids may be more efficiently improved by the assessment of always more patient feedback.

## 12 Excursion: Hearing loss and dementia

During the last years, another really important research branch came up regarding the clarification of hearing loss especially in higher ages: the correlation between cognitive abilities and losses and hearing impairment. The provision of hearing aids probably becomes more important in terms of prophylaxis and intelligence preservation [46].

### 12.1 Hearing loss in higher ages and mental abilities

It is well known that hearing loss impairs the ability of older people in road traffic, especially in cases of distraction, as a study group from Australia found out [84]. It investigated 107 people between 62 and 88 years. 55% of them had normal hearing abilities, 26% had a low-grade and 19% a moderate or severe hearing loss. The driving ability was measured on flat roads without distraction, with auditory, and with optic distraction signals. It could be observed that the driving abilities of hearing impaired people were significantly reduced with the presence of distraction signals compared to people with normal or only slightly impaired hearing.

Also the psychosocial health is influenced by hearing loss, which could be confirmed in a study of 1,178 older people from the Netherlands; hearing impaired people were mostly more lonesome [85].

### 12.2 Dementia and hearing loss

Already in 1989, Uhlmann and co-workers [86] compared 100 Alzheimer patients with a control group of the same size and indicated that a hearing loss of 30 dB and more seems to promote the development of Alzheimer’s disease. In a prospective trial from Baltimore [87], 639 individuals were followed-up for several years and regularly underwent examinations. Between 1990 and 1994, audiometric tests were performed; all participants were free of dementia signs at the beginning of the study. 71.2% of the people had normal hearing abilities, 19.6% had a low, 8.3% a moderate, and 0.9% a severe hearing loss. After an average follow-up period of 11.9 years, 58 cases of dementia were diagnosed (according to the consensus conference on diagnosis of dementia), among them 37 had Alzheimer’s disease. The risk to develop dementia was increased in cases of higher hearing loss. In comparison to normally hearing people, it was higher of 1.89 for low hearing loss, for moderate hearing loss it was threefold, and for severe hearing loss even 4.94-fold higher. Regarding the incidence of Alzheimer’s disease, the relation was also observed but the risk relation was lower.

In a recent population-related evaluation in Utah, USA, more than 4,400 persons who were more than 65 years old and had no dementia at the time of first examination, were examined exhaustively [88]. The average age was 75.4 years, by means of questionnaires and interviews the dementia status, the cognitive abilities, and the hearing (only formulated as question!) were assessed. After 10 years, 18.7% were dement compared to 12.1% of the unimpaired group. From a statistical point of view, hearing loss could thus be considered as significant predictor for developing dementia and the loss of cognitive abilities. However, the study does not reveal if the compensation of an existing hearing impairment (by hearing aids) had an influence on the risk development.

However, the problem of coincidence and direct causal correlation seems to be very important for future research; actually, the increasing age of society leads to a higher incidence of dementia, especially in the industrial nations. Hereby, the interrelationship of dementia and hearing loss is various. Because of the missing mental stimulation, hearing impaired people develop dementia possibly earlier, at the same time, however, the interaction with dement patients who are hearing impaired is clearly more difficult and also leads to increased agitation of dementia patients. Finally, it is also possible that people are considered as being dement even if they are “only” hearing impaired and cannot participate in normal conversations or react inadequately.

So it will become very important to perform audiometric examinations also in dementia patients in order to identify hearing deficits and to introduce rehabilitation. Even if such an audiometric examination is not always easy and according to an investigation in a US-American retirement home with dementia patients (n=307) only 5% tolerated the complete examination procedure, at least 70% could be partially examined [89]. In an own pilot study, we can confirm those results, however, it is necessary that also central hearing examinations are included into the diagnosis in order to be able to start an adequate hearing care or hearing therapy which is necessary for dement patients to use hearing aids [90].

First studies on the effect of hearing aids on mental abilities have meanwhile been presented, however, with low evidence. So 3,670 patients who were older than 65 years, were re-examined after 25 years. Initially, 137 had severe hearing loss, 1,139 had a moderate hearing impairment; after 25 years, significantly better mental state result were observed in those who used hearing aids [91]. 

Good hearing aid provision led to restructuring and measurable changes of the brain with regard to the processing of complex and in particular amplified signals [92].

In an investigation with 16 adult hearing impaired people, it could be confirmed that they mentally tire quickly. By means of clinically well adjusted hearing aids, this fatigue can be reduced and the effort of hearing decreased [93]. 

In a study from Nashville, USA, 16 hearing impaired people underwent a concentration test with and without hearing aids that took one hour [93]. The fatigue increased and the mental concentration decreased significantly when the tasks had to be fulfilled without hearing aids. Of course, the increasing mental fatigue was especially clear when the tasks consisted in speech perception. 

A study from Korea [94] evaluated the question if the use of hearing aids over a certain time improved speech understanding in noise and the cognitive speech-related functions. 18 participants with an average age of 69.5 years who were hearing impaired and used hearing aids, were compared to 11 hearing impaired people without hearing aids (average age of 63.1 years). The hearing aid users could significantly improve their short-term memory after 6 months, also the learning ability clearly increased. But even after 6 months, the audiometric tests (words in noise) were not significantly better compared to the control group. The authors conclude that the use of hearing aids influences the central cognitive functions and improves in particular the processing of speech perception. Hearing loss reduces the sensory integration and impairs cognitive functions; a provision of hearing aids may improve or even reverse this situation.

### Conclusion

Even in older patients, expectations regarding hearing aids are very high when they finally decide to use them. But then hearing aids cannot only improve the communication skills but the increased sensory stimulation even improves the cognitive, of course especially speech-related functions. Conversely, if not treated, hearing loss leads to the development of mental impairment. Further research activities on the significance of hearing aids regarding the reduction of mental abilities and the development of dementia are planned and will certainly be published. 

## 13 Special indications: Hearing aids in cases of tinnitus, hyperacusis, and noise sensitivity

According to the definition, hyperacusis is normal hearing but the patients seem to hear even “better than normal”. Because of the loud noise, they often develop typical vegetative anxiety reactions. In single cases, the habituation to noise is indicated with so-called noisers, however, there are currently no reliable studies on this topic. These devices are applied in the context of tinnitus retraining therapy (TRT) and also for hyperacusis, apparently with success, which cannot be explained by the noisers alone. An overview about this topic and a summary can be found in the articles published by Jastreboff [95], [96]. 

Hearing aids may be a very useful therapy for other types of noise sensitivity, such as especially the quasi physiological recruitment as result of inner ear hearing loss in the sense of functional loss of the outer hair cells. Also in this context, no reliable evaluations are found, only the clinical experience reveals that a compensation of an existing hearing loss by well and flexibly adjusted hearing aids reduces among others the central amplification effects for this frequency range and thus also the sensitivity for those frequencies [97].

### 13.1 Hearing aids for tinnitus

Since much more than 90% of the patients suffering from tinnitus are hearing impaired, nearly always in the frequency range of the tinnitus [98], the treatment of the diagnosed hearing loss with hearing aids is also important for tinnitus therapy, which is clinically undisputed. Often hearing aids lead to significant reduction of the tinnitus loudness.

Unfortunately, there are only few studies that examine the effectiveness of hearing aids on the tinnitus alone. Nearly always, the hearing aids are part of a combined therapy and cannot be evaluated in an isolated and solitary way. A recent Cochrane analysis confirms this aspect: One single study was found that compared the effect of hearing aids with noisers. It revealed the same (positive) effect for both approaches. All other studies mentioned a high effect of hearing aids but they were embedded in multimodal therapy concepts [99]. So also the recent S3 guideline on tinnitus [100] cannot recommend hearing aids in the context of tinnitus therapy even if the clinical experience confirms their benefit.

Indeed, this observation is a great problem: In up to 95%, tinnitus is associated with hearing loss and thus a symptom of impaired hearing perception. With hearing aids, many patients experience effective relief of their complaints, but studies on hearing aids as solitary therapy do not exist because they are always embedded in an audio-therapeutic concept. Nonetheless, hearing aids are essential in the tinnitus therapy because they nearly always include and compensate the existing hearing loss. As a consequence, cortical compensation reaction and a decrease of the inhibition are reduced.

Single studies emphasize this fact: 

In a retrospective tinnitus analysis [101], 58 tinnitus patients with hearing loss were followed-up; 29 used hearing aids, 29 did not – both groups had nearly identical audiograms, the same duration of tinnitus, and the same age on the average. Only in the group of hearing aid users, a significant improvement of the tinnitus could be achieved (based on the tinnitus handicap questionnaire, THQ).

A group from Marseille reported about 74 tinnitus patients who received hearing aids with a linear frequency transposition [102]. Those hearing aids are especially active in the high frequencies and very suitable for patients with a steep drop of the hearing curve. In 60 patients, the tinnitus could be permanently eliminated, 38 patients were examined more in detail: The majority of the patients (n=23) had tinnitus after noise exposition, the tinnitus suppression started few days after regular use of hearing aids and was permanent; however some days after non-use of the hearing aid, it was reduced and could be re-established after re-use. Further it was not dependent from the frequency of the tinnitus. The authors explain this phenomenon that is only achieved by the special transposition in the hearing aid, with a reactivation of deprived areas of the auditory cortex. This leads less to a direct stimulation but rather to an opening of neuronal canals (“gate mechanism”).

In Auckland, a special hearing aid feature was tested in 25 tinnitus patients to find out the influence on the tinnitus. Single frequencies were reduced and others were amplified [103]. Most suitable was a reduction of 6 dB at 2 kHz. The according software will be further evaluated and correlated with the tinnitus frequency. 

The principle of frequency transposition turned out to be effective in the hearing aid fitting for high frequency hearing loss with steep drop. Some manufacturers of hearing aids pursue this concept for many years, especially regarding children in order to enhance the speech level. Hereby, high frequencies are transposed and shifted to the middle frequencies and then provided via the hearing aid. Other manufacturers try to maximally amplify the high frequencies. Interestingly, a suppression of the tinnitus is successful in particular in tinnitus patients with high-frequency ear noise, however, it cannot be predicted for all patients. The French study confirms this phenomenon in all of their patients. Our own clinical experience shows similar effects also for the other approach. The reactivation of the tonotopic map of the auditory cortex leads to plastic changes that also influence the tinnitus, either by direct re-stimulation or a mediated gate effect.

A group from Berlin and London examined 15 tinnitus patients and found out that improvement of the tinnitus by hearing aids can only be achieved when the tinnitus frequency is located in the range of the actual transmission properties of the hearing aid, i.e. <6 kHz [104].

An overview from Netherland evaluated 786 articles, but only 10 could be identified as valid. Improvement was shown in 25–72% of the patients, the tinnitus was eliminated in 8–45% of the patients. Up to 25%, however, even mentioned deterioration, new additional tinnitus developed in up to 10%. But in summary, a high rate of improvements could be confirmed [105]. Another study evaluated 24 patients of 60–70 years of age. Hearing aid fitting improved the tinnitus loudness as well as the stress [106].

Often also combination devices are used for tinnitus therapy. Hereby, an additional noise or sound for relaxation may be added beside an amplification of the missing frequencies and thus compensation of the existing hearing loss is achieved. Those tinnitus devices may generally be prescribed (at the expense of the statutory health insurances), but there are no studies confirmed the usefulness of these devices. Further it must be questioned if a compensation of the existing hearing loss should be coupled with an additional noise because in this way central amplification effects are again activated especially because hearing is masked by the additional noise [98]. The use of a noiser alone with confirmed hearing loss is directly contraindicated because cortical enhancements emphasize the tinnitus frequency when hearing is made difficult due to additional noise.

An article from Iran reported about 974 war veterans that hearing aids are clearly better accepted than noise generators [107]. All patients had a significant hearing loss, 84% preferred a hearing aid, only 2% used noise generators, and 14% used both devices. The use of hearing aids led to a significant improvement of the tinnitus.

Finally it is the tinnitus itself that influences relevantly the course of the patients with tinnitus, as was confirmed by a group from Mannheim, Germany. They followed-up 28 patients with “fresh” tinnitus for 6 months [108]. At the beginning, a pure tone audiogram was measured, the tinnitus loudness and the noise sensitivity were assessed by analogue scales. During the 6 months, the tinnitus loudness as well as the stress remained constant while the noise sensitivity decreased. The initial depression as well as the hearing loss correlated with the higher tinnitus loudness. The authors postulate that the early detection of depressive components is as important for the improvement of the symptoms as the early fitting of hearing aids.

Also implantable hearing aids and cochlear implantations for severe hearing loss or deafness with tinnitus may have a positive effect on the tinnitus treatment. An overview of studies about the use of different hearing aids in the tinnitus therapy emphasized the particular importance of hearing loss compensation [109]. For hearing aids, the effect of tinnitus improvement is nearly 70%. For the special indication of treatment with implantable hearing aids in the context of tinnitus, there are actually not evaluations but the risk must always be considered that noise (drill) and surgical stress may generate new tinnitus. This fact generally applies for the provision of cochlear implants, but many studies report about predominantly positive effects of bilateral cochlear implantation of deaf or severely hearing impaired patients with regard to tinnitus. Recently, this is also reported for unilaterally deaf patients [110]. In summary, tinnitus is highly relevant for the quality of life and thus also for the results of CI patients [111]. Recently, a review article was published by the Charité in Berlin, Germany, that reported an improvement of the tinnitus by CI in 46–95% of the cases. The author states that not only the loudness should be assessed but also the psychological stress [112].

A study including 79 CI patients from Korea diagnosed tinnitus in 59 patients (74.7%). After cochlear implant surgery, the tinnitus was eliminated in 10 patients (25%), in 16 (40%) it was clearly improved. In summary, the results of the according questionnaires (THI and BDI) were significantly better 6 months after CI [113].

### Conclusion

Meanwhile modern digital hearing aids are very important in tinnitus therapy. Especially since an open, well tolerable provision of an external receiver, good feedback and noise suppression is also available for patients with high frequency hearing loss and high frequent tinnitus, a decisive progress was made. Those hearing aids also influence higher-frequency tinnitus because the transmission of those frequencies (up to 10 kHz) is now more effective. The use of additional noise for normal amplification should be well considered because the amplification is reduced or the patient is distracted which may lead to a reduced inhibition in the cortex and the auditory pathway and thus to increased tinnitus. All this must be taken into account in every single case.

Satisfactory hearing aids and thus rehabilitation of the hearing loss reduces the stress of hearing processing and leads to cortical re-structuring that may reduce tinnitus and also hyperacusis. Cochlear implantation for high-grade hearing loss and deafness is also useful especially because the hearing pathway is adequately stimulated which often reduces the tinnitus perception. But also in cases of unilateral deafness, the directional hearing may be optimized and tinnitus improvement is achieved. This development showed new ways because in particular unilateral deafness with torturing tinnitus could only be treated insufficiently with CROS or BAHA devices; generally the tinnitus could not be influenced. It is fascinating how cortical plasticity succeeds in integrating normal hearing in one ear with CI in the other and thus achieving a good hearing impression. Of course the extremely high costs must be considered but the improvement of the quality of life of the patients is a very high benefit.

## 14 Hearing protection and hearing aids for working in noise

In the broadest sense, also hearing protection is part of the group of hearing devices. This is especially true in the context of active hearing protection since many workers in noise and patients with (acknowledged) noise-induced hearing loss depend on hearing aids for improved communication or elimination of tinnitus. At the same time they have to protect themselves from noise exposure to avoid further deterioration of their hearing abilities. This aspect concerns working in noise and the necessary, even obligatory protection as well as noise to which people are exposed in their spare time, e.g. listening to music (discos, loud concerts) or other activities such as hunting, shooting, or loud motor noise. A particularly difficult situation arises for orchestra musicians who might need hearing aids as well as good hearing protection.

### 14.1 Working in noise

People who are regularly exposed to more than 85 dB (A) at their working place, have to use hearing protections that has to be provided by the employer. The requirements of hearing protection are defined by the DIN EN 352-2 regulation for Europe. Since there are different sensitivities of the skin of the auditory canal, hygienically well cleanable hearing protectors are available, either made of acryl or – even better tolerable – of silicone. The reduction of the environmental noise amounts to 22–26 dB according to the filter, while high frequencies are clearly more filtered (>30 dB). For special requirements, also differentiated filters may be adapted to the hearing protection. Another hearing protection that is even more effective (30 dB on the average) is achieved by the use of earmuffs. Nowadays they are less heavy (around 200 g), however, the worker has to get used to them and they are less tolerated in high temperatures. It is a particular problem for tinnitus patients to wear hearing protection because the tinnitus is perceived more loudly with the acoustic shielding. When they use hearing protection devices, hearing impaired patients will understand even less. For those persons, only an active hearing protection is appropriate even if it is more complex and of course expensive.

Such an active earmuff is equipped with 2 adjustable microphones, an internal electronic system reduces every noise exposure from 82 dB onwards; weak noise is amplified by 10, loud noise is reduced to the admitted measure. Such a hearing protection would also be recommended for musicians. Especially musicians in electronically amplified bands are exposed to significant noise levels but also orchestra musicians have to cope with high loudness levels in narrow and acoustically poorly isolated rehearsal rooms or orchestra pits. For those persons, individually adjusted (with otoplasty) hearing protection is available that is provided with several attenuation features (15, 20, and 25 dB). The above-mentioned active hearing protection could then be used in an even more effective and better way, however, because of the optic effect it will not prevail. But it was tested and primarily developed for rock bands.

Unfortunately, the general readiness of musicians to effectively protect their hearing organ that is so important for their profession, is only small, which could be shown by Richter et al. in a large survey from 2011 [114]. Perhaps, a rethinking occurs in this context, also due to the increasing number of job-related hearing losses for which compensations have to be paid that force especially the professional associations to undertake measures. Since re-constructions are often avoided because of the high expenses, the individual hearing protection has to be used. So musicians have to be ready to use it, at least for fortissimo passages, even if the musical control of the own performance is possibly more difficult.

### 14.2 Hearing protection in the leisure sector

Regarding the numerous noise exposures in the leisure sector, such as for example music in loud concerts, discos, certain sports events, and especially motor sport, but also for hunters and shooters, appropriate hearing protections are available, starting with very simple ones up to individually adjusted ones with possible attenuations of 15–30 dB. It is necessary that a conscious desire develops to really protect the own hearing and to avoid damage – this consciousness, however, sometimes only matures when already damage has occurred. This necessity exists especially for small children and infants when they are exposed to high loudness levels; it is recommended to use specially designed earmuffs designed for children with soft-touch materials, which can achieve a high attenuation without completely shielding from the environment.

## 15 Hearing training programs

In addition to hearing aid provision, hearing training programs have been developed in the last years, some of them have turned out to be useful, others, however, were less effective.

Generally, a review article in the context of geriatrics showed that the fitting of hearing aids always has to be individual and with particular consideration of the hearing habits, abilities, and special circumstances of the hearing impaired people [115]. This also means that first users of hearing aids are asked which sounds and noises are uncomfortable: from 60 participants of a Swedish study, 91% mentioned uncomfortable noises. Those noises have to be taken into account when adjusting the hearing aid. Often, fine-tuning of the hearing aids and also good information (generally habituation to those uncomfortable noise occurs quickly via the habituation effect) may improve the results [116].

From 26 older hearing aid users (average age of 79 years) who were not satisfied with their device, 80% benefitted from hearing training and targeted comparing adjustment [117].

Especially, spatial hearing with hearing aids can be significantly improved by targeted training, which could be shown in a controlled study from Illinois. 15 participants were allotted to 3 groups and examined regularly for 3 months by means of special tests for spatial resolution; 2 groups underwent training at home and in the hospital in alternating sequence, the third group did not receive training. The groups with hearing training had significantly better results in the tests compared to those without training [118].

A study from Australia suggested several methods for rehabilitation to 153 hearing impaired persons of a total of 289 examined patients: Besides hearing aid fitting, it was either communication training or no measure. 6 months after this consultation, 43% had received hearing aids, 18% had absolved the communication training program, and 39% had not undergone any measure. Predictors of successful rehabilitation were a higher socio-economic status, a severer initial impairment, and a higher willingness to change the situation. Physician should well support their patients in the decision of good hearing rehabilitation [119].

**Figure 5:**
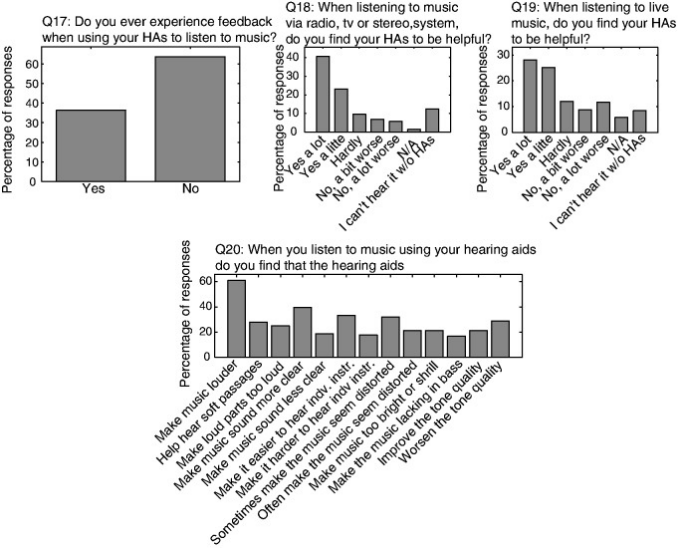
Questions on hearing aid use with the purpose of listening to music [120]

In the hearing and audio-therapies, often musical examples are applied successfully, however, there is a problem with regard to the use of hearing aids. Modern hearing aid technology focuses mainly on an improved speech understanding. Improved perception of music is taken less into consideration. An article from Cambridge asked (via internet) 523 hearing aid users regarding their hearing loss and their hearing habits. Most of them used the hearing aids also for listening to music, but often distortions, feedbacks, and override occurred (Figure 5 (Fig. 5)). The authors see a high potential for improvement in order to increase the use of hearing aids and the patients’ satisfaction [120].

Of course, an improved speech understanding is a relevant and important task of good hearing aid provision. Already for the quality of life and also for audiotherapies that accompany hearing aid fitting, enjoying music is essential; however, often hearing aids fail in this context or they are not suitably adjusted. Nonetheless, special programs and settings of current technology may lead to very good results also with regard to music, assuming that hearing care professionals and patients work on it together.

Recent studies on the effectiveness of hearing therapies are presented for example from a group from Korea: 20 older hearing aid users were evaluated, 10 of them underwent a 4-level training for 4 weeks (meaningless words) and 10 received a normal fitting. With the special hearing training, a significant improvement of the consonant and sentence test was revealed [121].

In a randomized and controlled study from Nottingham, 103 hearing aid users were trained by means of video-assisted interactive training units, 100 did not undergo additional therapy. The training took one hour every day for 6 weeks (on a voluntary basis), especially first users wore their device more frequently and benefitted significantly from this training [122].

In contrast, compared to a control group without training, no statistically significant difference regarding speech understanding in noise could be found in first hearing aid users who underwent hearing training via internet (USA). The conclusion of the authors was that such an internet-mediated training would be an interesting and cheap alternative comparable to expensive clinical training programs (that are, however, effective!) [123].

In Germany, partly very offensively promoted hearing training programs such as “Terzo” could not confirm the evidence of an additional effect. This is aggravated by the fact that the program was not allowed to be published due to patent protection and thus could not be verified even if it consisted of simplest hearing and writing items.

### Conclusion

Beside an improved and quality-controlled hearing aid provision, an accompanying hearing training is important. This fact is taught in the education of audio-therapists of the German association of hearing impaired people (Deutscher Schwerhörigenbund, DSB). Such therapy programs are available as manuals for everyone [15]. They definitely increase the acceptance of hearing aids and promote the timely and also spatial orientation, which was confirmed by some studies. Furthermore, training may reduce noise amplifications as uncomfortable perceived by habituation effects.

## 16 Conclusions

In the last years, hearing aid technology has progressed enormously and the possibilities of hearing rehabilitation have been clearly improved. Nonetheless, the provision of hearing aids is complex and not always satisfactory. It must be taken into account that device-related hearing care can never replace the normal hearing function and cannot lead to hearing improvement in every acoustic situation of life of a hearing impaired individual. Furthermore, hearing aid adjustment requires a habituation phase of up to several weeks in order to get used to a new hearing perception.

ENT specialist should not forget to undertake the tasks of good hearing rehabilitation. Neither hearing care professionals nor general practitioners, nor psychologists are in a position to responsibly and objectively fulfil these requirements. Compared to other European countries, the situation in Germany is better regarding the at least partial financing of hearing aids by the statutory health insurances, but the devices are sometimes very expensive, are associated with high financial expenses for the patient, and often consultation is not really adapted to the needs of the individual patient. That is why a good verification by the ENT specialist is absolutely necessary; it should be performed in a quality-controlled way as it is for example validated and published by the APHAB procedure.

Actually, it should no longer happen that ENT specialist tell their hearing impaired patients that they needed no hearing aid or were too young. In this way, a suitable care is shifted to a higher age and at the same time it becomes more and more difficult.

## Notes

### Competing interests

The authors declare that they have no competing interests.
